# Supplementary material for: The downward spiralling nature of the North Atlantic Subtropical Gyre
Source: Nat Commun. 2022 Apr 14;13:2000. doi: 10.1038/s41467-022-29607-8 (PMC9010438; doi:10.1038/s41467-022-29607-8)
Supplement: Supplementary file 1 — Supplementary Information [file 41467_2022_29607_MOESM1_ESM.pdf]

Supplementary Material
The Downward Spiralling Nature of the North Atlantic Subtropical Gyre

Sara Berglund[1,*], Kristofer Döös[1], Sjoerd Groeskamp[2], and Trevor J. McDougall[3]

[1]Department of Meteorology, Stockholm University, Stockholm, Sweden
[2]NIOZ Royal Netherlands Institute for Sea Research, Den Burg, Texel, The Netherlands
[3]School of Mathematics and Statistics, University of New South Wales, Australia
[*]sara.berglund@misu.su.se

March 23, 2022

Supplementary Table 1: Values are computed from trajectories simulated with the eddy-permitting model, with a horizontal resolution of 1/4° and 75 depth levels. The total volume transport (percentage of the total volume transport reaching north) (1 Sv= 10^6 m^3 s^{-1}), average heat change, average salt change, average density increase, average depth increase and average circuit time for all waters in each circuit of the Gyre. The heat (salt) change describes how much heat (salt) all parcels have lost/gained for one specific circuit. The depth and density increase shows how much the depth and density increase by average for all waters in each circuit of the Gyre. The average time of each circuit shows in general how long it takes for waters to follow that specific circuit. The volume transport and also heat, salt, density and depth change for each circuit are in agreement with the lower resolution case (1°). However, one circuit takes about half the time to finish in the higher resolution, which is discussed more in the main text.

Circuit	Volume transport [Sv] (%)	Heat [TW]	Salt [10^6 kg/s]	$\Delta\sigma_0$ [kg/m^3]	Δz [m]	Time [years]
1	7.8	-111	-0.75	0.75	112	14
2	7.1	-48	-0.78	0.20	55	25
3	4.9	-25	-0.51	0.09	36	34
4	3.4	-14	-0.30	0.05	26	40
5	2.4	-9	-0.20	0.04	21	43
6	1.7	-6	-0.13	0.03	16	45
7	1.3	-4	-0.09	0.01	8	46

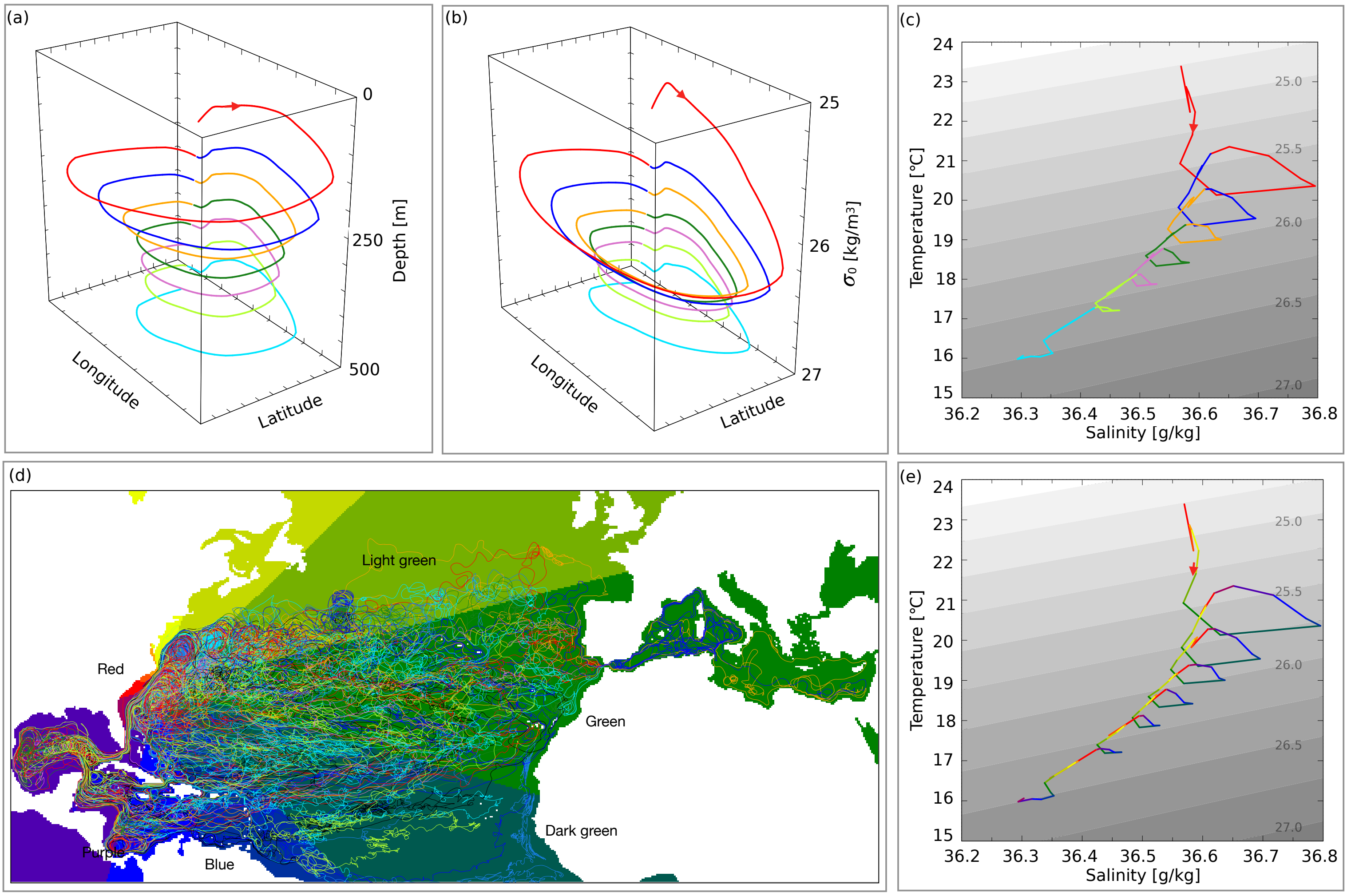

Supplementary Figure 1: The Downward Spiral computed from trajectories simulated with the eddy-permitting model with a horizontal resolution of 1/4° and 75 depth levels. A mean trajectory computed from all trajectories that goes through seven or more circuits in the Gyre. Each circuit is coloured with one colour except for e, which is coloured following the colour–clock in d. (a): 3D illustration of the average trajectory in depth, latitude and longitude coordinates. (b): 3D illustration of the average trajectory in σ_0, latitude and longitude coordinates. (c): The average trajectory in temperature-salinity space. Grey contours are σ_0-surfaces. (d): A set of trajectories simulated in the present study that circuits the Gyre. Only their circulation in the Gyre is plotted. The colour wheel is used to compute the average trajectory in a,b,c and e. The names of the colours are included as they are discussed in the text. (e): The average trajectory coloured after location following the colour-clock superimposed in d.

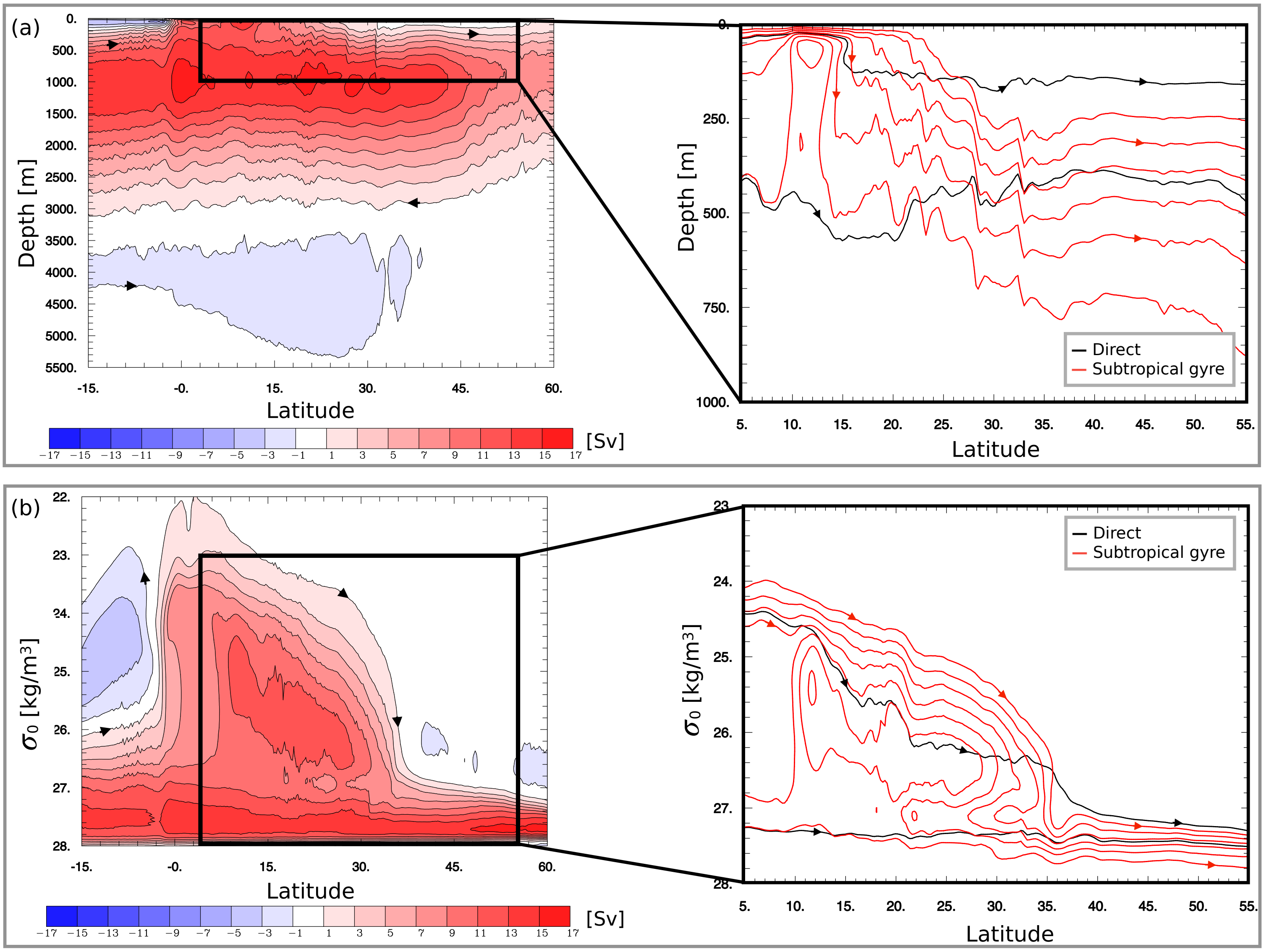

Supplementary Figure 2: The Atlantic Meridional Overturning circulation in the eddy-permitting model with a horizontal resolution of 1/4° and 75 depth levels. (a) Left: The Eulerian Atlantic Meridional Overturning stream function computed for the years 1950 - 2005. The rectangle indicates the area shown in the figure to the right. The contour interval is 2 Sv. Right: The Lagrangian Meridional Overturning stream function computed from trajectories simulated with the eddy-permitting model and only those that move directly northwards without spiralling in the Gyre (black contours) and for those waters that spiral in the Gyre at least once (red contours). The contour interval is 1 Sv. (b) Left: The Eulerian Atlantic Meridional Overturning stream function in σ_0-latitude coordinates computed for the same period as in a. The black rectangle indicates the region shown in the right panel. The contour interval is 2 Sv. Right: The Lagrangian Meridional Overturning stream function in σ_0-latitude coordinates for waters that move directly northwards without spiralling in the Gyre (black contours) and for those waters that spiral in the Gyre at least once (red contours). The contour interval is 1 Sv.

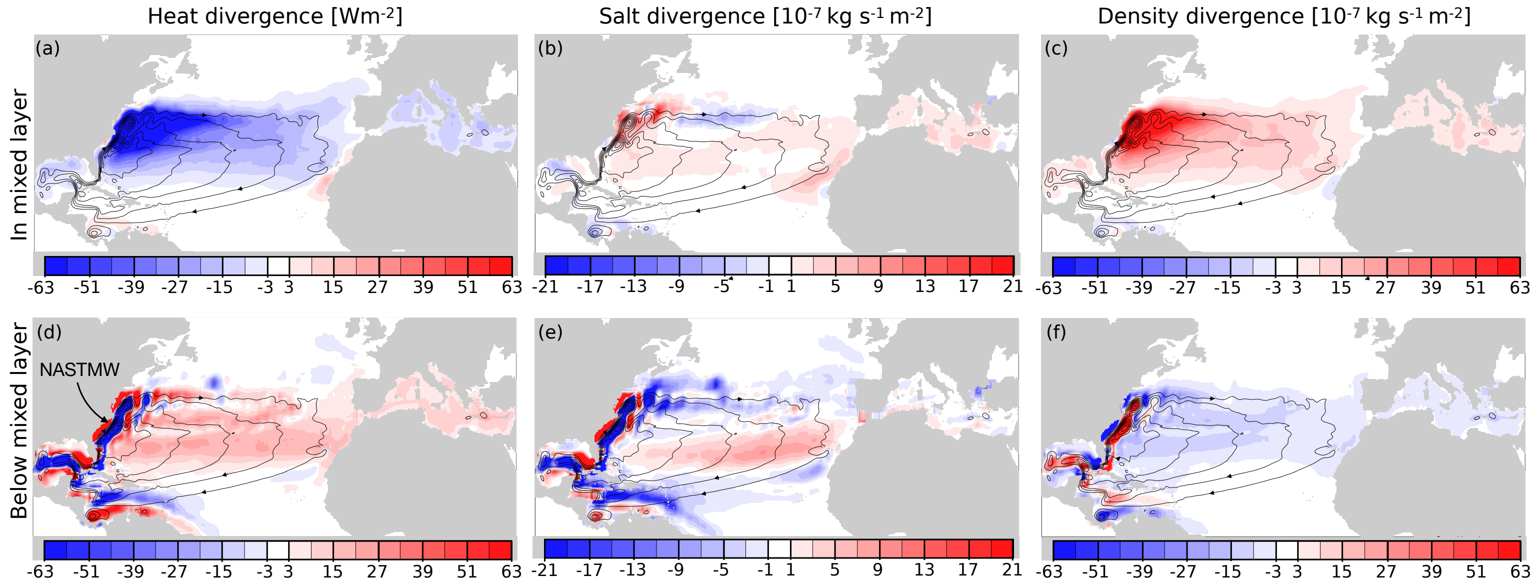

Supplementary Figure 3: The Lagrangian divergence computed with trajectories simulated with data from the eddy-permitting model. Upper panel: The Lagrangian divergences of trajectories that spiral the Gyre in the mixed layer. Superimposed on all figures are the Lagrangian Barotropic stream function computed only for the Gyre circulation. The contour interval of the stream function is 2 Sv. (a)-(c): The heat, salt and density divergence in the mixed layer computed for the trajectories circuiting the subtropical gyre. Negative values indicate a loss of heat, salt or density. Lower panel: The Lagrangian divergences below the mixed layer depth of the trajectories that spiral in the Gyre. As in the upper panel the Lagrangian Barotropic stream function is superimposed, with contour intervals of 2 Sv. (d)-(f): The heat, salt and density divergences below the mixed layer for all trajectories circulating the Gyre. Negative values show a loss, whereas positive values indicate a gain. In d, e, and f three regions are marked as they are discussed in the text as the main regions where changes in heat, salt and density occurs below the mixed layer. The formation region of the North Atlantic Subtropical Mode Water (NASMW) is marked in d.
